# The Degree of Prepregnancy Vitamin D Deficiency Is Not Associated With Gestational Diabetes in Women Undergoing ART

**DOI:** 10.1210/jendso/bvad140

**Published:** 2023-11-10

**Authors:** Yvonne Liu, Johann-Georg Hocher, Huijun Chen, Liang Hu, Xiaoli Zhang, Sufen Cai, Sha Tang, Fei Gong, Bernhard K Krämer, Ge Lin, Berthold Hocher

**Affiliations:** Fifth Department of Medicine (Nephrology/Endocrinology/Rheumatology/Pneumology), University Medical Centre Mannheim, University of Heidelberg, 68167 Mannheim, Germany; Medical Faculty of Charité Universitätsmedizin Berlin, 10117 Berlin, Germany; Fifth Department of Medicine (Nephrology/Endocrinology/Rheumatology/Pneumology), University Medical Centre Mannheim, University of Heidelberg, 68167 Mannheim, Germany; Second Faculty of Medicine, Charles University, 150 06 Prague, Czech Republic; Fifth Department of Medicine (Nephrology/Endocrinology/Rheumatology/Pneumology), University Medical Centre Mannheim, University of Heidelberg, 68167 Mannheim, Germany; Medical Faculty of Charité Universitätsmedizin Berlin, 10117 Berlin, Germany; Department of Clinical Science, Reproductive and Genetic Hospital of CITIC-Xiangya, Changsha 410008, China; Department of Clinical Science, Reproductive and Genetic Hospital of CITIC-Xiangya, Changsha 410008, China; School of Basic Medical Science, Institute of Reproductive and Stem Cell Engineering, Central South University, Changsha 410017, China; Key Laboratory of Stem Cells and Reproductive Engineering, Ministry of Health, Changsha 410017, China; Fifth Department of Medicine (Nephrology/Endocrinology/Rheumatology/Pneumology), University Medical Centre Mannheim, University of Heidelberg, 68167 Mannheim, Germany; Institute of Pharmacy, Freie Universität Berlin, 14195 Berlin, Germany; Department of Clinical Science, Reproductive and Genetic Hospital of CITIC-Xiangya, Changsha 410008, China; School of Basic Medical Science, Institute of Reproductive and Stem Cell Engineering, Central South University, Changsha 410017, China; Department of Clinical Science, Reproductive and Genetic Hospital of CITIC-Xiangya, Changsha 410008, China; Department of Clinical Science, Reproductive and Genetic Hospital of CITIC-Xiangya, Changsha 410008, China; School of Basic Medical Science, Institute of Reproductive and Stem Cell Engineering, Central South University, Changsha 410017, China; Key Laboratory of Stem Cells and Reproductive Engineering, Ministry of Health, Changsha 410017, China; Fifth Department of Medicine (Nephrology/Endocrinology/Rheumatology/Pneumology), University Medical Centre Mannheim, University of Heidelberg, 68167 Mannheim, Germany; Key Laboratory of Stem Cells and Reproductive Engineering, Ministry of Health, Changsha 410017, China; Department of Clinical Science, Reproductive and Genetic Hospital of CITIC-Xiangya, Changsha 410008, China; School of Basic Medical Science, Institute of Reproductive and Stem Cell Engineering, Central South University, Changsha 410017, China; Key Laboratory of Stem Cells and Reproductive Engineering, Ministry of Health, Changsha 410017, China; Fifth Department of Medicine (Nephrology/Endocrinology/Rheumatology/Pneumology), University Medical Centre Mannheim, University of Heidelberg, 68167 Mannheim, Germany; Department of Clinical Science, Reproductive and Genetic Hospital of CITIC-Xiangya, Changsha 410008, China; School of Basic Medical Science, Institute of Reproductive and Stem Cell Engineering, Central South University, Changsha 410017, China; Institute of Medical Diagnostics, IMD, 12247 Berlin, Germany

**Keywords:** vitamin D, free vitamin D, gestational diabetes, in vitro fertilization

## Abstract

**Context:**

Gestational diabetes mellitus (GDM) is a common pregnancy complication, particularly in women undergoing assisted reproductive technology (ART). An association of GDM with vitamin D in women conceiving naturally has been described; however, studies have yielded heterogeneous results.

**Objective:**

To analyze the association between prepregnancy total and free vitamin D and GDM incidence in women undergoing ART.

**Methods:**

Post hoc analysis of a prospective study at the Reproductive and Genetic Hospital of CITIC-Xiangya in Changsha, China. Total and free vitamin D were measured 1 day before embryo transfer. The patients were screened for GDM using the oral glucose tolerance test.

**Results:**

A total of 1593 women were included in the study, among whom 256 (16.1%) developed GDM. According to international guidelines for total 25-hydroxyvitamin D [25(OH)D], 47 (2.9%) patients had sufficient (≥30 ng/mL) levels, while 696 (43.7%) were insufficient (20 to <30 ng/mL) and 850 (54.4%) were deficient (<20 ng/mL). Comparing GDM and non-GDM patients, there was no significant difference in total nor free vitamin D levels (*P* = .340 and .849). Similarly, analysis of GDM rates by quintiles of total and free 25(OH)D showed no significant association in one-way ANOVA (*P* = .831 and .799). Multivariate logistic regression, considering age, BMI, and fasting glucose, also did not show a significant influence of the 2 vitamin D forms on GDM incidence (*P* = .266 and .123 respectively).

**Conclusion:**

In this relatively vitamin D deficient/insufficient ART cohort, the degree of neither total nor free vitamin D deficiency before pregnancy was associated with the occurrence of GDM.

Vitamin D deficiency is widespread in the general population, including pregnant women, in different regions of the world (1, 2). In line with this, multiple studies have shown the importance of vitamin D during pregnancy and that a deficiency could be associated with gestational diseases and complications, such as gestational diabetes (GDM) and preeclampsia (2). However, clinical studies on this topic are limited and present inconsistent results.

GDM is defined as diabetes that is diagnosed during pregnancy and was not present before. It is one of the most common pregnancy complications, with a prevalence of 14.8% in China (2019) (3), meaning that together with its large population, China has among the largest number of GDM patients worldwide. A group that is particularly at risk are women who undergo assisted reproductive technologies (ART), that is, undergoing in vitro fertilization (IVF) and/or intracytoplasmic sperm injection (ICSI) (4, 5). At the same time, the use and development of these procedures have been rising rapidly in the past decades, enabling many people with infertility problems to have children. As the diagnosis also has acute and long-term consequences for both mother and child (6), it is important to evaluate the different risk factors associated with GDM to prevent its occurrence as best and early as possible. In this study, we will analyze if prepregnancy vitamin D status has an impact on the incidence of GDM and can be classified as an independent risk factor for women undergoing ART.

To evaluate a patient's vitamin D status, the commonly used clinical parameter is total 25-hydroxyvitamin D [25(OH)D]. This measurement comprises 3 forms of vitamin D: more than 99% is protein-bound, either to vitamin D–binding protein (DBP) (about 85%) or to albumin (about 15%). Only a very small amount (<1%) is unbound and free (7, 8). The problem with measuring total 25(OH)D is that many factors, such as estrogen concentration and liver function (8), influence the concentration of the main transport protein DBP, which in turn leads to fluctuations in total 25(OH)D. This is where the interest in “free vitamin D” has been rising in recent years. Free 25(OH)D is independent of factors that affect DBP concentration, which potentially makes it a more suitable parameter to assess vitamin D status, particularly in certain cohorts, such as pregnant women (9). Although it is not yet a routinely used parameter, it may be a better indicator of vitamin D status, which is why we measured both in this study.

Because women who undergo IVF/ICSI have an increased risk for GDM, it is especially important to study additional risk factors that are associated with the incidence of GDM in this cohort. At present, the number of clinical studies on the relationship between vitamin D and GDM is limited and the results are heterogeneous (10). In addition, there are no studies on the possible effect of vitamin D status on GDM in women undergoing ART. Therefore, the purpose of this study is to assess the relationship between vitamin D, both total and free 25(OH)D, and GDM in this specific cohort.

## Methods

### Study Design

This study is a post hoc analysis of a prospective single-center clinical study. The primary study was concerned with the effect of vitamin D status on IVF/ICSI outcome (11). Data were collected between January 2017 and December 2018 at the Reproductive and Genetic Hospital of CITIC-Xiangya in Changsha, China. The study was approved by the ethics committee of the hospital (approval number: LL-SC-2018-014) and was carried out with the written, informed consent of all participants.

### Study Cohort

From 3600 initial patients considered eligible for the study, the final cohort of the primary study included 2569 women, who underwent fresh embryo transfer at the hospital during the study period. However, among women who got pregnant, data about the incidence of GDM were only available for 1593 patients, who comprised the final cohort for this study. These women received IVF/ICSI treatment, followed by fresh embryo transfer, as already described in the study protocol (11, 12).

Inclusion criteria were: (1) age from 18 to 40 years; (2) first IVF/ICSI treatment; (3) received IVF/ICSI treatment, as well as fresh embryo transfer; (4) informed consent was given by the patient for participation in the study; and (5) clinical pregnancy diagnosed. The exclusion criteria were (#1 to #11 of the following criteria were screened for every patient before treatment): (1) received oocyte donation; (2) uterine malformation; (3) endometriosis; (4) uterine adhesions; (5) untreated hydrosalpinx; (6) uterine myoma; (7) Cushing syndrome; (8) adult-onset adrenogenital syndrome; (9) hypothalamic or pituitary disease causing infertility; (10) diabetes mellitus type 1 or 2 prior to pregnancy; (11) hypertension prior to pregnancy (blood pressure values above 140 mmHg systolic or 90 mmHg diastolic; or taking antihypertensives); and (12) incomplete data regarding the incidence of GDM.

### Data Collection

Total and free vitamin D were measured before pregnancy, 1 day before embryo transfer, but after hormonal stimulation, both using a commercially available enzyme-linked immunosorbent assay (ELISA) kit (DIAsource ImmunoAssays: Total, RRID: AB_3068001; Free, RRID: AB_2890998). Free vitamin D levels can be obtained directly or indirectly through calculation. In this study, it was measured directly, which is more accurate (8). According to the Endocrine Society, total vitamin D levels are classified as follows: (1) vitamin D sufficiency (≥30 ng/mL); (2) vitamin D insufficiency (20 to <30 ng/mL); and (3) vitamin D deficiency (<20 ng/mL) (13, 14). However, there is no set range for total 25(OH)D during exceptional circumstances, such as pregnancy, as well as for specific ethnic groups. Likewise, there are no reference levels for free 25(OH)D to date. Suggested values for free 25(OH)D by Zeng et al are 5.67 pg/mL [equivalent to 20 ng/mL total 25[OH]D] and 8.50 pg/mL [equivalent to 30 ng/mL total 25[OH]D] (15).

Concerning the outcomes, this paper focuses on a secondary endpoint of the primary study: the incidence of GDM. During pregnancy, all study patients were screened for gestational diabetes using a 75-g oral glucose tolerance test, as previously described (12). Gestational diabetes was diagnosed if it was not present prior to pregnancy, and blood glucose levels were in one of the following categories: (1) ≥92 mg/dL (5.1 mmol/L) before glucose intake; (2) ≥180 mg/dL (10.0 mmol/L) 1 hour after glucose intake; or (3) ≥153 mg/dL (8.5 mmol/L) 2 hours after glucose intake, following the International Association of Diabetes in Pregnancy Study Groups (IADPSG) 2010 guidelines (12, 16). Patients were contacted by phone during the pregnancy and after delivery to follow up on further complications and outcomes.

### Statistical Analysis

Primary data was analyzed using SPSS version 29.0 (SPSS Inc., Chicago, IL, USA). Diagrams were created with GraphPad Prism 6 (GraphPad Software, San Diego, CA, USA). All study participants from the original study were included in this analysis if data regarding GDM was available. Therefore, we did not perform a sample size calculation. In the descriptive statistics, values are expressed either as frequency (%) or median (interquartile range [IQR]). The Chi-square (χ^2^) test for categorical variables and the Mann-Whitney U test for continuous variables were carried out to compare GDM and non-GDM patient groups. One-way analysis of variance (ANOVA) was used to compare GDM occurrence in the different vitamin D quintiles. We also created a multivariate logistic regression model, considering factors that had a significant *P* value in the descriptive statistics. For this analysis, we created 2 models: Model A, considering significant factors in literature: maternal age, obesity (via body mass index [BMI]), and prediabetes (via blood sugar measurement prior to fertility treatments). Model B included significant factors from Table 1 (maternal age, BMI, blood sugar, anti-Müllerian hormone [AMH], luteinizing hormone [LH], and estradiol). All patients were of Chinese descent, while other risk factors, such as a family history of diabetes, were not recorded. *P* < .05 was considered statistically significant.

**Table 1.** Characterization of the study population—binary analysis

Parameters	All (*n* = 1593)	GDM (*n* = 256)	No GDM (*n* = 1337)	*P* value
Age, years	29.0 (27.0-31.0)	30.0 (28.0-32.0)	29.0 (27.0-31.0)	<.001
BMI, kg/m^2^	21.33 (19.64-23.05)	22.19 (20.41-23.68)	21.22 (19.57-22.90)	<.001
Hypertension	61 (3.8%)	47 (3.5%)	14 (5.5%)	.136
Systolic RR, mmHg	115 (108-121)	115 (109-121)	115 (108-121)	.571
Diastolic RR, mmHg	76 (70-81)	75 (70-81)	76 (70-81)	.778
Blood sugar, mmol/L	5.15 (4.92-5.41)	5.23 (5.00-5.53)	5.13 (4.90-5.39)	<.001
Total 25(OH)D, ng/mL	19.59 (16.61-22.92)	19.55 (16.51-22.64)	19.61 (16.67-23.00)	.**340**
Free 25(OH)D, pg/mL	4.72 (4.11-5.34)	4.76 (4.08-5.34)	4.72 (4.12-5.34)	.**849**
Infertility type				.980
1°	891 (55.9%)	143 (55.9%)	748 (55.9%)	
2°	702 (44.1%)	113 (44.1%)	589 (44.1%)	
Fertilization method				.243
IVF	1094 (68.7%)	184 (71.9%)	910 (68.1%)	
ICSI	262 (16.4%)	33 (12.9%)	229 (17.1%)	
IVF + ICSI	237 (14.9%)	39 (15.2%)	198 (14.8%)	
Birth outcome				.373
Full-term	1296 (81.5%)	208 (81.3%)	1088 (81.6%)	
Preterm	274 (17.2%)	48 (18.8%)	226 (16.9%)	
Early abortion	8 (0.5%)	0 (0.0%)	8 (0.6%)	
Late abortion	11 (0.7%)	0 (0.0%)	11 (0.8%)	
Ectopic pregnancy	1 (0.1%)	0 (0.0%)	1 (0.1%)	
Delivery method				.095
Normal	440 (27.6%)	76 (29.7%)	364 (27.2%)	
Cesarean	1131 (71.0%)	180 (70.3%)	951 (71.1%)	
AMH, ng/mL	5.77 (3.69-9.28)	5.09 (3.38-8.58)	5.88 (3.75-9.47)	.027
FSH, mIU/mL	5.58 (4.78-6.54)	5.48 (4.62-6.50)	5.61 (4.78-6.54)	.266
LH, mIU/mL	3.63 (2.63-5.00)	3.32 (2.39-4.56)	3.68 (2.68-5.09)	.002
Estradiol, pg/mL	33.00 (27.00-43.76)	31.00 (25.00-40.75)	34.00 (27.00-44.00)	<.001
Testosterone, ng/mL	0.28 (0.23-0.36)	0.29 (0.22-0.36)	0.28 (0.23-0.36)	.615
AFC	15.00 (12.00-21.00)	15.00 (12.00-20.00)	15.00 (12.00-22.00)	.274

Data in the table are shown as frequency, *n* (%) for categorical variables, or median (IQR) for continuous variables. Comparisons were made using the Chi-square (χ^2^) test for categorical variables and Mann-Whitney U test for continuous variables.

Abbreviations: 25(OH)D, 25-hydroxyvitamin D; AFC, antral follicle count; AMH, anti-Müllerian hormone; BMI, body mass index; FSH, follicle-stimulating hormone; GDM, gestational diabetes mellitus; ICSI, intracytoplasmic sperm injection; IVF, in vitro fertilization; LH, luteinizing hormone; RR, blood pressure.

## Results

In accordance with the criteria mentioned in “Methods,” a total of 1593 patients undergoing embryo transfer after IVF/ICSI were included in this study. The median age was 29 years (IQR, 27-31). These patients were grouped into GDM and non-GDM patients for binary analysis (Table 1). A total of 256 (16.1%) of the 1593 patients were diagnosed with GDM during their pregnancy. Age, BMI, and blood sugar measured prior to embryo transfer showed a statistically significant difference between the 2 groups (*P* < .05), as did the hormones AMH, LH, and estradiol. Contrastingly, neither total nor free 25(OH)D was statistically significantly different (*P* = .340 and .849 respectively) when comparing GDM and non-GDM patients.

**Figure 1.**
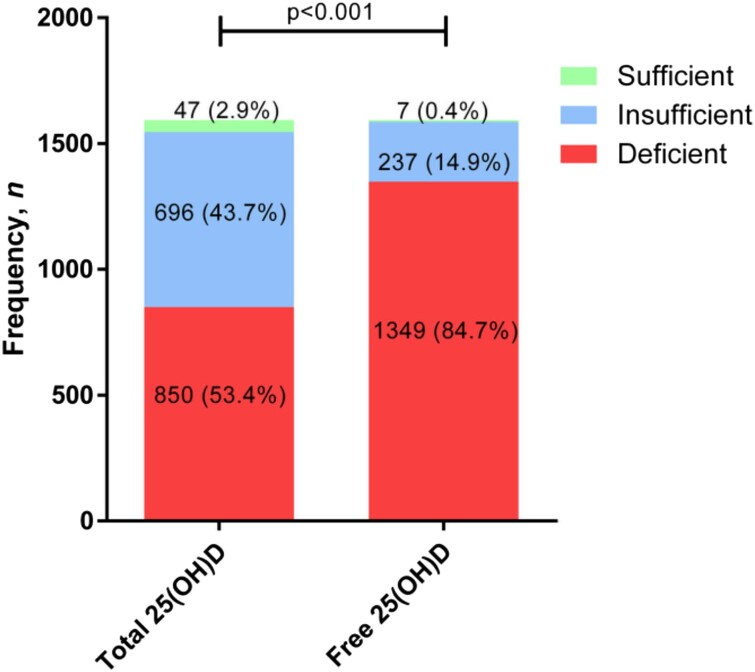
Distribution of study population in total and free 25-hydroxyvitamin D [25(OH)D] categories. Data are shown as frequency, *n* (%). Comparison was made with the Chi-square (χ^2^) test. Total 25(OH)D was classified into 3 groups as follows: deficiency (<20 ng/mL), insufficiency (20 to <30 ng/mL), and sufficiency (≥30 ng/mL). Free 25(OH)D was classified into 3 groups using the values suggested by Zeng et al (15): deficiency (<5.67 pg/mL) insufficiency (5.67 to <8.50 pg/mL), and sufficiency (≥8.50 pg/mL).

Following the clinical practice guidelines of the Endocrine Society for total vitamin D (13), only 47 (2.9%) patients had a sufficient total 25(OH)D supply, according to the guidelines; 696 (43.7%) patients had an insufficient and 850 (53.4%) had a deficient vitamin D status (Fig. 1).

For free vitamin D, there are no internationally established reference values. Using the suggested values from Zeng et al (15), only 7 (0.4%) study participants had a sufficient supply of free 25(OH)D of more than 8.50 pg/mL. Another 237 (14.9%) were insufficient and the large majority, 1349 (84.7%), were deficient (Fig. 1).

**Figure 2.**
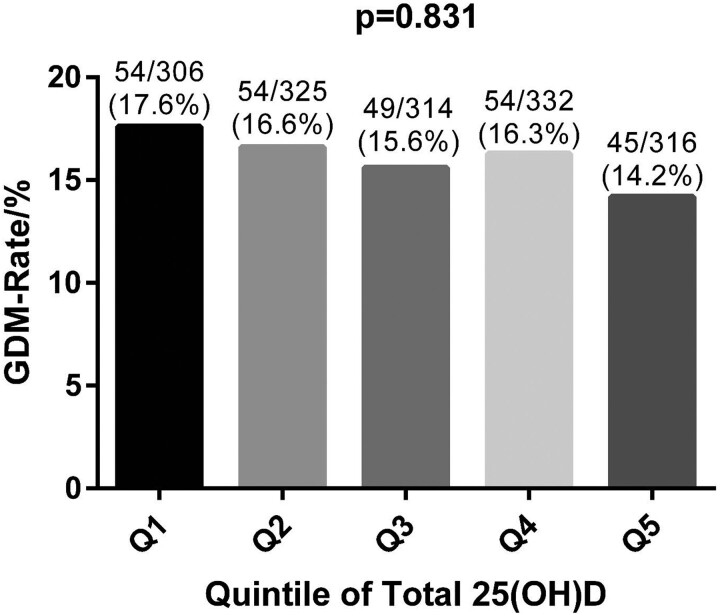
Comparison of GDM rates by quintiles of total 25(OH)D. Comparisons were made using one-way ANOVA. Quintiles of total 25(OH)D were defined in ng/mL as follows: Q1 (<15.94), Q2 (15.94 to <18.50), Q3 (18.50 to <20.67), Q4 (20.67 to <23.80), and Q5 (≥23.80). Abbreviations: 25(OH)D, 25-hydroxyvitamin D; GDM, gestational diabetes mellitus.

**Figure 3.**
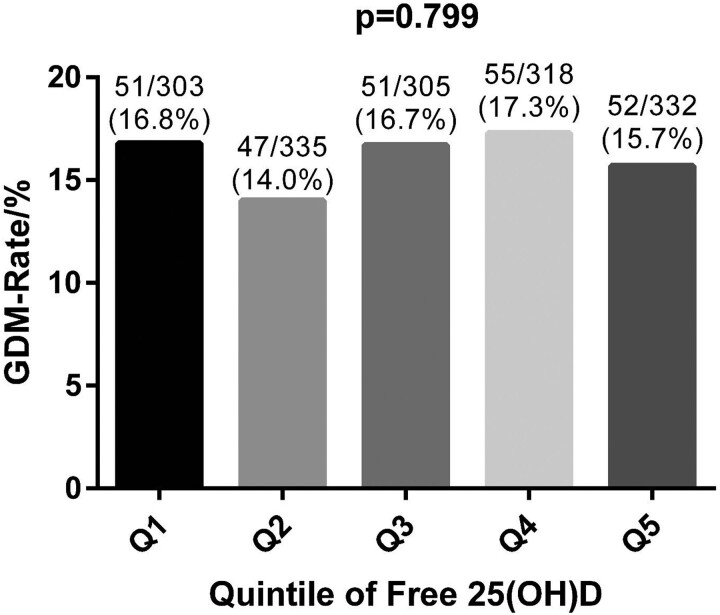
Comparison of GDM rates by quintiles of free 25(OH)D. Comparisons were made using one-way ANOVA. Quintiles of free 25(OH)D were defined by concentration/pg/mL as follows: Q1 (<3.97), Q2 (3.97 to <4.48), Q3 (4.48 to <4.96), Q4 (4.96 to <5.48), and Q5 (≥5.48). Abbreviations: 25(OH)D, 25-hydroxyvitamin D; GDM, gestational diabetes mellitus.

The study group was divided into quintiles, separately according to total and free vitamin D, to analyze the incidence of GDM in each of the quintiles. One-way ANOVA showed no significant difference in GDM incidence in the different quintiles for both total and free 25(OH)D (*P* = .831 and .799 respectively) (Figs. 2 and 3).

To further explore the association between vitamin D and GDM in this relatively vitamin D insufficient/deficient population, we used multivariate logistic regression, taking into account confounding factors. Factors in Model A are established risk factors for GDM from the available literature, namely, maternal age, BMI, and blood sugar prior to fertility treatments. Considering these factors, there was no statistical association between the degree of either total or free 25(OH)D deficiency with the incidence of GDM, with a *P* value of .266 and .123, respectively (Tables 2 and 3). For Model B, factors that were significantly different between GDM and non-GDM patients (from Table 1) were included in the analysis: maternal age, BMI, blood sugar, AMH, LH, and estradiol (Tables 4 and 5). Similarly, this multivariate logistic regression also showed no significant association (*P* = .297 and .094 respectively) (Tables 4 and 5).

**Table 2.** Multivariate logistic regression for total 25(OH)D and GDM incidence (model A)

	B	OR	95% CI	Standard error	*P* value
Total 25(OH)D	−.016	0.984	[0.955, 1.013]	0.015	.**266**
Age	.013	1.120	[1.075, 1.167]	0.021	<.001
BMI	.092	1.096	[1.035, 1.160]	0.029	.002
Blood sugar	.409	1.505	[1.107, 2.047]	0.157	.009

Comparisons were made using multivariate logistic regression.

Abbreviations: 25(OH)D, 25-hydroxyvitamin D; B, regression coefficient; BMI, body mass index; GDM, gestational diabetes mellitus; OR, odds ratio.

**Table 3.** Multivariate logistic regression for free 25(OH)D and GDM incidence (model A)

	B	OR	95% CI	Standard error	*P* value
Free 25(OH)D	0.102	1.107	[0.973, 1.261]	0.066	.**123**
Age	0.111	1.118	[1.073, 1.164]	0.021	<.001
BMI	0.095	1.100	[1.039, 1.165]	0.029	.001
Blood sugar	0.423	1.527	[1.122, 2.078]	0.157	.007

Comparisons were made using multivariate logistic regression.

Abbreviations: 25(OH)D, 25-hydroxyvitamin D; B, regression coefficient; BMI, body mass index; GDM, gestational diabetes mellitus; OR, odds ratio.

**Table 4.** Multivariate logistic regression for total 25(OH)D and GDM incidence (model B)

	B	OR	95% CI	Standard error	*P* value
Total 25(OH)D	−0.016	0.985	[0.956, 1.014]	0.015	.**297**
Age	0.011	1.117	[1.072, 1.164]	0.021	<.001
BMI	0.086	1.090	[1.029, 1.155]	0.030	.004
Blood sugar	0.401	1.493	[1.097, 2.032]	0.157	.011
AMH	−0.018	0.982	[0.951, 1.014]	0.016	.268
LH	0.005	1.005	[0.962, 1.051]	0.023	.816
Estradiol	−0.010	0.028	[0.982, 0.999]	0.004	.028

Comparisons were made using multivariate logistic regression.

Abbreviations: 25(OH)D, 25-hydroxyvitamin D; AMH, anti-Müllerian hormone; B, regression coefficient; BMI, body mass index; GDM, gestational diabetes mellitus; LH, luteinizing hormone; OR, odds ratio.

**Table 5.** Multivariate logistic regression for free 25(OH)D and GDM incidence (model B)

	B	OR	95% CI	Standard error	*P* value
Free 25(OH)D	0.112	1.118	[0.981, 1.275]	0.067	.**094**
Age	0.109	1.115	[1.070, 1.162]	0.021	<.001
BMI	0.090	1.094	[1.033, 1.160]	0.030	.002
Blood sugar	0.414	1.513	[1.110, 2.061]	0.158	.009
AMH	−0.017	0.983	[0.952, 1.015]	0.016	.291
LH	0.007	1.007	[0.962, 1.054]	0.023	.778
Estradiol	−0.010	0.990	[0.981, 0.998]	0.004	.021

Comparisons were made using multivariate logistic regression.

Abbreviations: 25(OH)D, 25-hydroxyvitamin D; AMH, anti-Müllerian hormone; B, regression coefficient; BMI, body mass index; GDM, gestational diabetes mellitus; LH, luteinizing hormone; OR, odds ratio.

## Discussion

### Short Summary of Results

Most of the 1593 women taking part in this study did not have a sufficient vitamin D supply. In this relatively vitamin D insufficient/deficient cohort of women undergoing IVF/ICSI and embryo transfer, the degree of both total and free vitamin D deficiency before pregnancy was not associated with the incidence of GDM.

### Interpretation of Results

A total of 256 (16.1%) patients developed GDM, which reflects the high incidence of this pregnancy complication. This is also in accordance with the meta-analysis by Gao et al, which found that the GDM prevalence in China was fairly high at 14.8% in 2019 (3).

The present study also confirms the high prevalence of vitamin D deficiency. Prior to their pregnancy, most patients were either vitamin D insufficient (43.7%) or even deficient (53.4%), totaling 97.1% of the patients. Therefore, only 47 (2.9%) had a sufficient total 25(OH)D supply (≥30 ng/mL), according to current international general guidelines for vitamin D status (13).

Almost all patients in the study population had a lack of vitamin D, which is very common in China, although the percentages vary across the country. Studies in Beijing (17) and Guizhou (18) found a very high percentage of vitamin D deficiency among pregnant women (total 25(OH)D < 20 ng/mL), of 96.8% and 92.8%, respectively. Other studies also found a fairly high prevalence of vitamin D insufficiency/deficiency (total 25(OH)D < 30 ng/mL) of 65.9% in Liuzhou (19) and 97.4% (of whom 57.5% were vitamin D deficient) in Chengdu (20), which is similar to the findings of our study. Important factors influencing vitamin D status in China include vitamin D supplementation, a lack of sunlight exposure due to a high level of indoor activities, and dietary habits (17, 21).

Furthermore, only 7 (0.4%) patients had sufficient free 25(OH)D levels according to the suggested cutoff of 8.50 pg/mL suggested by Zeng et al (15). This shows that there were even fewer patients with sufficient *free* 25(OH)D levels, compared to the percentage of patients with a sufficient *total* 25(OH)D supply (as seen in Fig. 1). Considering that the measurements were made before embryo transfer, but *after* hormonal stimulation, a possible explanation could be that the increase in estrogen due to hormonal stimulation led to an increase in DBP synthesis (8). Because more vitamin D would then be bound to its transport protein DBP, the proportion of free and bioavailable vitamin D would decrease. This is the first study that includes measurements of total *and* free vitamin D to evaluate its relationship with GDM. Therefore, we cannot compare the measurements of free 25(OH)D to other similar studies.

With such a small proportion of the study cohort having a sufficient supply of total and free vitamin D, the findings of this study focus on a vitamin D insufficient/deficient population. In this population, statistical analyses showed that the degree of vitamin D deficiency is not associated with GDM incidence—for both total and free vitamin D. Taking into account confounders, multivariate logistic regression also indicates that there is no association in this cohort between total or free 25(OH)D and GDM (*P* = .266 and .123 in Model A and *P* = .297 and .094 in Model B) (Tables 2 to 5). In other words, the degree of vitamin D deficiency does not affect the incidence of GDM. However, given the low number of women with sufficient vitamin D levels, clear conclusions on whether higher levels of vitamin D would have decreased the risk of GDM cannot be made.

Comparing our results to previous studies on the association between total 25(OH)D and GDM incidence in non-ART pregnancies, these studies present heterogeneous results. Meta-analyses by Rizzo et al (10) and Wei et al (22) suggested that there is an uncertain relationship between vitamin D and GDM, whereby some studies present a significant association (23, 24) and others show similar results to our study (25-27). We also reviewed similar studies in the last 10 years in a Chinese/Taiwanese population (Supplementary Table S1 (28)). While all 9 studies agree that there is a high prevalence of vitamin D deficiency, 4 of them found a statistically significant relationship between vitamin D and GDM (22, 29-31). One of the studies found that vitamin D was neither associated with GDM nor fasting glucose in Chinese women (32), while the other 4 studies show associations only under certain conditions. Three of them suggest that there is a J-shaped relationship (33) rather than a linear relationship, that is, that there is only a significant association with GDM above a certain level of vitamin D (>20 ng/mL (33, 34) or >30 ng/mL (35)). Another study found that vitamin D was only associated with GDM in the second but not the first trimester of pregnancy. Because most women in our study cohort were vitamin deficient/insufficient, the findings fit to the latter studies. In any case, it is noteworthy to mention that all above-mentioned studies were not conducted in women undergoing IVF/ICSI treatment, a procedure that increases GDM risk.

The strengths of this study include that it is a post hoc analysis of an originally prospective, clinical study with a large cohort of the same ethnic background. This reduced an important confounding factor, which is often a problem in large-scale meta-analyses. Furthermore, it is the first study on the association between prepregnancy vitamin D and GDM in women who undergo ART, which additionally includes *free* vitamin D.

A limitation that remains to be established is that there are no widespread guidelines for vitamin D levels for different sexes, ethnicities, or pregnant women, which is very important, as this study solely includes Chinese women. Because it is such a specific cohort, the results are not directly comparable to other study populations. Additionally, vitamin D measurements were made prior to pregnancy, 1 day before embryo transfer. For further analysis, one could measure vitamin D levels throughout the pregnancy, thereby also comparing how total and free vitamin D levels progress during pregnancy, and possibly influence the incidence of GDM. Additional steps for future studies on this topic are to take into account more quantitative data, such as the results of the oral glucose tolerance test. Furthermore, data on the family history of diabetes, which is an important risk factor for diabetes, was not available. It would also be useful to measure more factors, such as insulin resistance, which play a role in the genesis of (gestational) diabetes.

In our study, vitamin D measurements were made before pregnancy, which is a unique opportunity in IVF/ICSI patients, as it is possible to study the effects of a certain factor before pregnancy even occurs. This is the first study to investigate the effects of prepregnancy vitamin D concentrations on GDM, which would allow for the prevention of diseases. However, the measurements were made after hormonal stimulation and although free 25(OH)D is known to not be influenced as much as total 25(OH)D, more studies are needed at this point in time to evaluate the reliability.

It is a strength of this study that we analyzed both free and total vitamin D, especially given that total vitamin D concentrations might be affected by estrogen after ovarian stimulation for egg retrieval. However, the methods used are based on ELISA technology. Measurements based on liquid chromatography–mass spectrometry (LC-MS) would be more accurate but are very expensive and were not available for the study. This represents a clear study limitation. In the studies that Rizzo et al (10) reviewed, a correlation between total 25(OH)D and GDM was only found in studies that used LC-MS assays. However, from the 9 studies in Supplementary Table S1 (28), 2 used LC-MS and one of them also found that there was no statistical association (32); similarly, studies using immunoassays also found an association (29, 30).

This study has used a variety of statistical methods, 2 different types of vitamin D determinations, and 2 different regression models, which agree that there is no statistical association between vitamin D and GDM incidence in this cohort. This shows that despite different approaches and independent analysis methods, the result is the same in this cohort.

In conclusion, in this prospective study in Changsha, China, with women undergoing IVF/ICSI, the incidence of GDM was not associated with the degree of vitamin D deficiency/insufficiency before pregnancy—for both total and free vitamin D.

## Data Availability

Some or all datasets generated during and/or analyzed during the current study are not publicly available but are available from the corresponding author on reasonable request.

## Abbreviations

25(OH)D: 25-hydroxyvitamin D
AMH: anti-Müllerian hormone
ANOVA: analysis of variance
ART: assisted reproductive technology
DBP: vitamin D–binding protein
ELISA: enzyme-linked immunosorbent assay
GDM: gestational diabetes mellitus
ICSI: intracytoplasmic sperm injection
IQR: interquartile range
IVF: in vitro fertilization
LC-MS: liquid chromatography–mass spectrometry
LH: luteinizing hormone

## References

[1] Karras S, Paschou SA, Kandaraki E, et al Hypovitaminosis D in pregnancy in the Mediterranean region: a systematic review. Eur J Clin Nutr. 2016;70(9):979‐986. 26931671 10.1038/ejcn.2016.12

[2] Xiao JP, Zang J, Pei JJ, Xu F, Zhu Y, Liao XP. Low maternal vitamin D status during the second trimester of pregnancy: a cross-sectional study in Wuxi, China. PLoS One. 2015;10(2):e0117748. 25659105 10.1371/journal.pone.0117748 PMC4320063

[3] Gao C, Sun X, Lu L, Liu F, Yuan J. Prevalence of gestational diabetes mellitus in mainland China: a systematic review and meta-analysis. J Diabetes Investig. 2019;10(1):154‐162. 10.1111/jdi.12854 PMC6319492 29683557

[4] Ashrafi M, Gosili R, Hosseini R, Arabipoor A, Ahmadi J, Chehrazi M. Risk of gestational diabetes mellitus in patients undergoing assisted reproductive techniques. Eur J Obstet Gynecol Reprod Biol. 2014;176:149‐152. 24630294 10.1016/j.ejogrb.2014.02.009

[5] Wang J, Liu Q, Deng B, Chen F, Liu X, Cheng J. Pregnancy outcomes of Chinese women undergoing IVF with embryonic cryopreservation as compared to natural conception. BMC Pregnancy Childbirth. 2021;21(1):39. 33422044 10.1186/s12884-020-03486-7 PMC7796545

[6] Schafer-Graf UM, Gembruch U, Kainer F, et al Gestational diabetes Mellitus (GDM)—diagnosis, treatment and follow-up. Guideline of the DDG and DGGG (S3 level, AWMF registry number 057/008, February 2018). Geburtshilfe Frauenheilkd. 2018;78(12):1219‐1231. 30651660 10.1055/a-0659-2596 PMC6301211

[7] von Websky K, Hasan AA, Reichetzeder C, Tsuprykov O, Hocher B. Impact of vitamin D on pregnancy-related disorders and on offspring outcome. J Steroid Biochem Mol Biol. 2018;180:51‐64. 29169993 10.1016/j.jsbmb.2017.11.008

[8] Tsuprykov O, Chen X, Hocher CF, Skoblo R, Lianghong Y, Hocher B. Why should we measure free 25(OH) vitamin D? J Steroid Biochem Mol Biol. 2018;180:87‐104. 29217467 10.1016/j.jsbmb.2017.11.014

[9] Tsuprykov O, Buse C, Skoblo R, Haq A, Hocher B. Reference intervals for measured and calculated free 25-hydroxyvitamin D in normal pregnancy. J Steroid Biochem Mol Biol. 2018;181:80‐87. 29567112 10.1016/j.jsbmb.2018.03.005

[10] Rizzo G, Garzon S, Fichera M, et al Vitamin D and gestational diabetes Mellitus: is there a link? Antioxidants (Basel). 2019;8(11):511. 31731439 10.3390/antiox8110511 PMC6912234

[11] Cai S, Li J, Zeng S, et al Impact of vitamin D on human embryo implantation-a prospective cohort study in women undergoing fresh embryo transfer. Fertil Steril. 2021;115(3):655‐664. 33039126 10.1016/j.fertnstert.2020.09.005

[12] Chen H, Li J, Cai S, et al Blastocyst transfer: a risk factor for gestational diabetes Mellitus in women undergoing in vitro fertilization. J Clin Endocrinol Metab. 2022;107(1):e143‐e152. 34415990 10.1210/clinem/dgab594 PMC8684461

[13] Holick MF, Binkley NC, Bischoff-Ferrari HA, et al Evaluation, treatment, and prevention of vitamin D deficiency: an endocrine society clinical practice guideline. J Clin Endocrinol Metab. 2011;96(7):1911‐1930. 21646368 10.1210/jc.2011-0385

[14] Amrein K, Scherkl M, Hoffmann M, et al Vitamin D deficiency 2.0: an update on the current status worldwide. Eur J Clin Nutr. 2020;74(11):1498‐1513. 31959942 10.1038/s41430-020-0558-y PMC7091696

[15] Zeng S, Chu C, Doebis C, von Baehr V, Hocher B. Reference values for free 25-hydroxy-vitamin D based on established total 25-hydroxy-vitamin D reference values. J Steroid Biochem Mol Biol. 2021;210:105877. 33741448 10.1016/j.jsbmb.2021.105877

[16] International Association of Diabetes and Pregnancy Study Groups Consensus Panel, Metzger BE, Gabbe SG, et al International association of diabetes and pregnancy study groups recommendations on the diagnosis and classification of hyperglycemia in pregnancy. Diabetes Care. 2010;33(7):676‐682. 20190296 10.2337/dc09-1848 PMC2827530

[17] Song SJ, Zhou L, Si S, et al The high prevalence of vitamin D deficiency and its related maternal factors in pregnant women in Beijing. PLoS One. 2013;8(12):e85081. 24386450 10.1371/journal.pone.0085081 PMC3873449

[18] Hong-Bi S, Yin X, Xiaowu Y, et al High prevalence of vitamin D deficiency in pregnant women and its relationship with adverse pregnancy outcomes in Guizhou, China. J Int Med Res. 2018;46(11):4500‐4505. 30270806 10.1177/0300060518781477 PMC6259374

[19] Li X, Wang Y, Gao G, Guan X, Qin P, Liu C. High prevalence of vitamin D deficiency in pregnant women in south China. Int J Vitam Nutr Res. 2020;90(3-4):273‐278. 31188079 10.1024/0300-9831/a000592

[20] Wang J, Yang F, Mao M, Liu DH, Yang HM, Yang SF. High prevalence of vitamin D and calcium deficiency among pregnant women and their newborns in Chengdu, China. World J Pediatr. 2010;6(3):265‐267. 20706824 10.1007/s12519-010-0224-x

[21] Liu W, Hu J, Fang Y, Wang P, Lu Y, Shen N. Vitamin D status in mainland of China: a systematic review and meta-analysis. EClinicalMedicine. 2021;38:101017. 34308318 10.1016/j.eclinm.2021.101017 PMC8283334

[22] Chen GD, Pang TT, Li PS, et al Early pregnancy vitamin D and the risk of adverse maternal and infant outcomes: a retrospective cohort study. BMC Pregnancy Childbirth. 2020;20(1):465. 32795269 10.1186/s12884-020-03158-6 PMC7427871

[23] Soheilykhah S, Mojibian M, Rashidi M, Rahimi-Saghand S, Jafari F. Maternal vitamin D status in gestational diabetes mellitus. Nutr Clin Pract. 2010;25(5):524‐527. 20962313 10.1177/0884533610379851

[24] Zhang C, Qiu C, Hu FB, et al Maternal plasma 25-hydroxyvitamin D concentrations and the risk for gestational diabetes mellitus. PLoS One. 2008;3(11):e3753. 19015731 10.1371/journal.pone.0003753 PMC2582131

[25] Baker AM, Haeri S, Camargo CA Jr, Stuebe AM, Boggess KA. First-trimester maternal vitamin D status and risk for gestational diabetes (GDM) a nested case-control study. Diabetes Metab Res Rev. 2012;28(2):164‐168. 21818838 10.1002/dmrr.1282 PMC4381548

[26] Clifton-Bligh RJ, McElduff P, McElduff A. Maternal vitamin D deficiency, ethnicity and gestational diabetes. Diabet Med. 2008;25(6):678‐684. 18544105 10.1111/j.1464-5491.2008.02422.x

[27] Farrant HJ, Krishnaveni GV, Hill JC, et al Vitamin D insufficiency is common in Indian mothers but is not associated with gestational diabetes or variation in newborn size. Eur J Clin Nutr. 2009;63(5):646‐652. 18285809 10.1038/ejcn.2008.14 PMC2678985

[28] Liu Y, Hocher JG, Chen H, et al Supplementary table for: the degree of pre-pregnancy vitamin D deficiency is not associated with gestational diabetes in women undergoing ART. Figshare; 2023. doi: 10.6084/m9.figshare.24270592.v1 PMC10681737 38024652

[29] Shang M, Zhao N. Early pregnancy vitamin D insufficiency and gestational diabetes mellitus. J Obstet Gynaecol Res. 2022;48(9):2353‐2362. 35830973 10.1111/jog.15333

[30] Wang Y, Li H, Zheng M, et al Maternal vitamin D deficiency increases the risk of adverse neonatal outcomes in the Chinese population: A prospective cohort study. PLoS One. 2018;13(4):e0195700. 29689109 10.1371/journal.pone.0195700 PMC5915779

[31] Xu C, Ma HH, Wang Y. Maternal early pregnancy plasma concentration of 25-hydroxyvitamin D and risk of gestational diabetes Mellitus. Calcif Tissue Int. 2018;102(3):280‐286. 29058058 10.1007/s00223-017-0346-4

[32] Loy SL, Lek N, Yap F, et al Association of maternal vitamin D Status with glucose tolerance and caesarean section in a multi-ethnic Asian cohort: the growing up in Singapore towards healthy outcomes study. PLoS One. 2015;10(11):e0142239. 26571128 10.1371/journal.pone.0142239 PMC4646602

[33] Pham TTM, Huang YL, Chao JC, et al Plasma 25(OH)D concentrations and gestational diabetes Mellitus among pregnant women in Taiwan. Nutrients. 2021;13(8):2538. 34444700 10.3390/nu13082538 PMC8398607

[34] Yin WJ, Tao RX, Hu HL, et al The association of vitamin D status and supplementation during pregnancy with gestational diabetes mellitus: a Chinese prospective birth cohort study. Am J Clin Nutr. 2020;111(1):122‐130. 31625576 10.1093/ajcn/nqz260

[35] Cheng Y, Chen J, Li T, et al Maternal vitamin D status in early pregnancy and its association with gestational diabetes mellitus in Shanghai: a retrospective cohort study. BMC Pregnancy Childbirth. 2022;22(1):819. 36335302 10.1186/s12884-022-05149-1 PMC9636619

